# Bacterial polysaccharides—A big source for prebiotics and therapeutics

**DOI:** 10.3389/fnut.2022.1031935

**Published:** 2022-11-03

**Authors:** Raees Khan, Muhammad Dawood Shah, Luqman Shah, Ping-Chin Lee, Imran Khan

**Affiliations:** ^1^Department of Biological Sciences, National University of Medical Sciences, Rawalpindi, Pakistan; ^2^Borneo Marine Research Institute, Universiti Malaysia Sabah, Kota Kinabalu, Sabah, Malaysia; ^3^Department of Biochemistry, Faculty of Biological and Health Sciences, Hazara University, Mansehra, Pakistan; ^4^Biotechnology Research Institute, Universiti Malaysia Sabah, Kota Kinabalu, Sabah, Malaysia; ^5^Faculty of Science and Natural Resources, Universiti Malaysia Sabah, Kota Kinabalu, Sabah, Malaysia; ^6^Department of Biotechnology, Faculty of Chemical and Life Sciences, Abdul Wali Khan University Mardan, Mardan, Pakistan

**Keywords:** polysaccharides, bacteria, prebiotics, therapeutics, gut microbiota, anti-cancer, anti-inflammatory, antioxidant

## Abstract

Bacterial polysaccharides are unique due to their higher purity, hydrophilic nature, and a finer three-dimensional fibrous structure. Primarily, these polymers provide protection, support, and energy to the microorganism, however, more recently several auxiliary properties of these biopolymers have been unmasked. Microbial polysaccharides have shown therapeutic abilities against various illnesses, augmented the healing abilities of the herbal and Western medicines, improved overall health of the host, and have exerted positive impact on the growth of gut dwelling beneficial bacteria. Specifically, the review is discussing the mechanism through which bacterial polysaccharides exert anti-inflammatory, antioxidant, anti-cancer, and anti-microbial properties. In addition, they are holding promising application in the 3D printing. The review is also discussing a perspective about the metagenome-based screening of polysaccharides, their integration with other cutting-edge tools, and synthetic microbiome base intervention of polysaccharides as a strategy for prebiotic intervention. This review has collected interesting information about the bacterial polysaccharides from Google Scholar, PubMed, Scopus, and Web of Science databases. Up to our knowledge, this is the first of its kind review article that is summarizing therapeutic, prebiotics, and commercial application of bacterial polysaccharides.

## Introduction of microbial polysaccharides

One of the main categories of carbohydrates are polysaccharides which are composed of monosaccharide units as their building blocks. Among them, D-glucose is the most predominant compound. In addition to glucose, some other derivatives of monosaccharides are also found in polysaccharides that include simple sugar acids such as glucuronic and iduronic acid, amino sugars like D-galactosamine, D-glucosamine, and their derivatives like N-acetylneuraminic acid and N-acetylmuramic acid (1). These polysaccharides come from various sources such as algae (e.g., alginate), plants (e.g., pectin and gums), bacteria (e.g., dextran), and animals (e.g., chitosan). Besides their function as structural material, polysaccharides are also considered important functional material, playing a variety of roles in many physiological and biological processes. They display anti-tumor, anti-hyperglycemic, and immune modulatory potentials (2) (Table 1).

**Table 1:** Sources, type, and potential properties/applications of some well-known bacterial polysaccharides.

**Producer bacterial species**	**Name of the polysaccharide**	**The activity of the polysaccharide**	**References**
*Acetobacter polysaccharogenes*	Acetan	Antiallergic	(173, 174)
*Acetobacter xylinum, Pseudomonas* sp., *Agrobacterium Rhizobium*	Cellulose	Tissue-engineered blood vessels and wound dressings	(175)
*Alcaligenes, Halomonas*	EPSA	Antibacterial	(176)
*Bacillus thuringiensis*	EPSA	Antioxidant/ anti-cancer	(96)
*Bacillus amyloliquefaciens*	EPSA	Antioxidant/ anti-cancer	(95)
*Bacillus subtilis, Streptococcus mutans, Streptococcus salivarius, Gluconacetobacter xylinus, Zymomonas mobilis*	Levan		(175)
*B. licheniformis* B3-15	EPSA	Antiviral	(177, 178)
*Enterobacter* sp., *Clavibacter* sp., and *Klebsiella* sp.	Fucose containing exopolysaccharides (FcEPSs)		(179)
*Enterobacter cloacae*	Exopolysaccharide	Anti-cancer (cervical) activity	(180, 181)
*Escherichia coli*	Heparan sulfate mimetics derived from K5 polysaccharide (K5PSs)	Anti-tumor activity (angiogenesis inhibition)	(182)
*Geobacillus* sp. WSUCF1	EPSA	Antioxidant	(183)
*Geobacillus thermodenitrificans* B3-72	EPSA	Immunomodulatory and antiviral	(184)
*Lactobacillus acidophilus*	EPSA	Anti-cancer	(106)
*Lactobacillus hilgardii, L. rhamnosus, L. kefir, L. kefiranofascien*	Kefiran	Anti-tumor potential	(185)
*Pasteurella multocida* and *Streptococcus* sp.	Hyaluronic acid [N-acetyl glucosamine and Glucuronate linked through β-(1,4) linkages]	Tissue repair, drug delivery, cosmetics, and viscosupplementation	(186)
Rhizobium sp. N613	EPSA	Anti-tumor	(104)
*Rhizobium* sp., *Cellulomonas* spp., and *Agrobacterium* sp.	Curdlan [Glucose molecules joined by β-(1,3) linkages]	Food additive	(187)
*Streptomyces virginia* H03	EPSA	Antioxidant	(92)
*Polaribacter* sp.	EPSA	Antioxidant	(98)
*Pseudomonas* sp. WAK-1	EPSA	Anti-cancer	(105)
*Pseudomonas aeruginosa* and *Azotobacter vinelandii*	Alginate	Antioxidant	(188, 189)
*Xanthomonas* sp.	Xanthan (Glucose Mannose, Glucuronic Acid, Acetate Pyruvate)	Food additive, cosmetics, and agriculture	(190)
*Sphingomonas paucimobilis ATCC 31461*	Gellan (Glucose Rhamnose, Glucuronic Acid, Acetate, and Glycerate)	Food & feed, gel electrophoresis	(191)
*Pseudomonas stutzeri* 273	EPSA	Antioxidant, anti-biofouling	(192, 193)
*Bacillus altitudinis* MSH2014	EPSA	anti-cancer	(194)

Microbial polysaccharides are high molecular weight carbohydrates produced by microorganisms such as bacteria, fungi, yeast, and algae. These polysaccharides include carbohydrates that are produced and accumulated inside the cells such as glycogen where they function as energy and carbon reserves. Others include polysaccharides of cell wall such as chitin, involved in stabilization of the integrity of the cells, and extracellular polysaccharides secreted by the cells. The later type either form a capsule (capsular polysaccharides) that remains associated with the surface of the cell or a slime loosely attached to the surface of the cell (exopolysaccharides) (3). Capsular polysaccharides are usually associated with pathogenicity and virulence promoting factors of bacteria, whereas exopolysaccharides are mostly responsible to cope with the environmental stress, adhere to the surfaces and serve as water and carbon reservoirs (4). Exopolysaccharides (EPSs) are usually built by monosaccharide units and non-carbohydrate components such as acetate, phosphate, pyruvate, and succinate. EPSs are further divided into two groups: homopolysaccharides and heteropolysaccharides. Homopolysaccharides such as cellulose, dextran, and pullulan are composed of only one type of monosaccharide units, however, heteropolysaccharides such as xanthan, and hyaluronic acid contain two or more than two kind of monosaccharides (5). Bacteria produce EPSs to protect themselves against phagocytosis and environmental stress. Lactic acid producing bacteria are well-known for EPSs production (6). *Weissella confuse, Lactobacillus* spp., Pediococcus spp. and *Leoconostoc* spp. produce EPSs that exhibit immunomodulatory and antioxidant activities (7).

In association with other biological molecules such as lipids, proteins, and nucleotides, polysaccharides are an integral part of the biological systems and perform several functions, like cellular communications, surface adhesion, and molecular recognition by the components of the immune system. These biopolymers also possess therapeutic abilities against tumor, provide protection against various insults, and responses to a variety of stimuli (8, 9). The applications of microbial polysaccharides in biotechnology and biomedical sciences were initiated. As these polysaccharides are derived from diverse sources, which makes them the best option to be used as a starting material that can be easily modified and then employed for various purposes, such as food production, energy generation, in the preparation of wood, paper, textiles, fibers, and in drilling of oil (10, 11). Polysaccharides also has the potential to be utilized as carriers of anti-cancer drugs, which has attained great attention (12). Polysaccharides of fungal origin may be used as a multi-purpose prescription in the coming years (13). It has been found that some well-known polysaccharides such as dextran and levans were developed and polymerized in the exterior environment of the cells, where the naturally occurring enzymes were present that transformed substrates into their polymeric forms.

Polysaccharides differ from each other based on monosaccharide compositions, length of the chain, and the extent of their branching system. There are several instances, where a polysaccharide family produced by microbes has a significant role in food processing industry. Various factors such as composition of the media, pH, temperature, and the presence/absence of air, may have a significant impact on production of microbial polysaccharides as they play an important role in the physiological processes of the microbes (14).

## Types of microbial polysaccharides and their applications

Microbial polysaccharides exhibit a great diversity in their chemical composition and structures. Compared to plants, microbial polysaccharides are more suitable for large scale production. Microbes also offer great diversity in terms of their polysaccharides as different genera belonging to a particular class are capable to produce specific polysaccharides. The biological properties of microbial polysaccharides are dependent on the type of monosaccharide units, composition, molecular weight, and extent of branching, as well as the chemistry of the polysaccharides. For example, hydrophobic and low molecular weight polysaccharides exhibit less antitumor potential as compared to their counterparts. On the other hand, polysaccharides containing β-(1 → 3) glycosidic bonds in their backbone structure, and β-(1 → 6) branching points, show superior anticancer activities (15). Before discussing microbial polysaccharides, we first discuss some of the well-known microbial monosaccharides.

**Figure 1:**
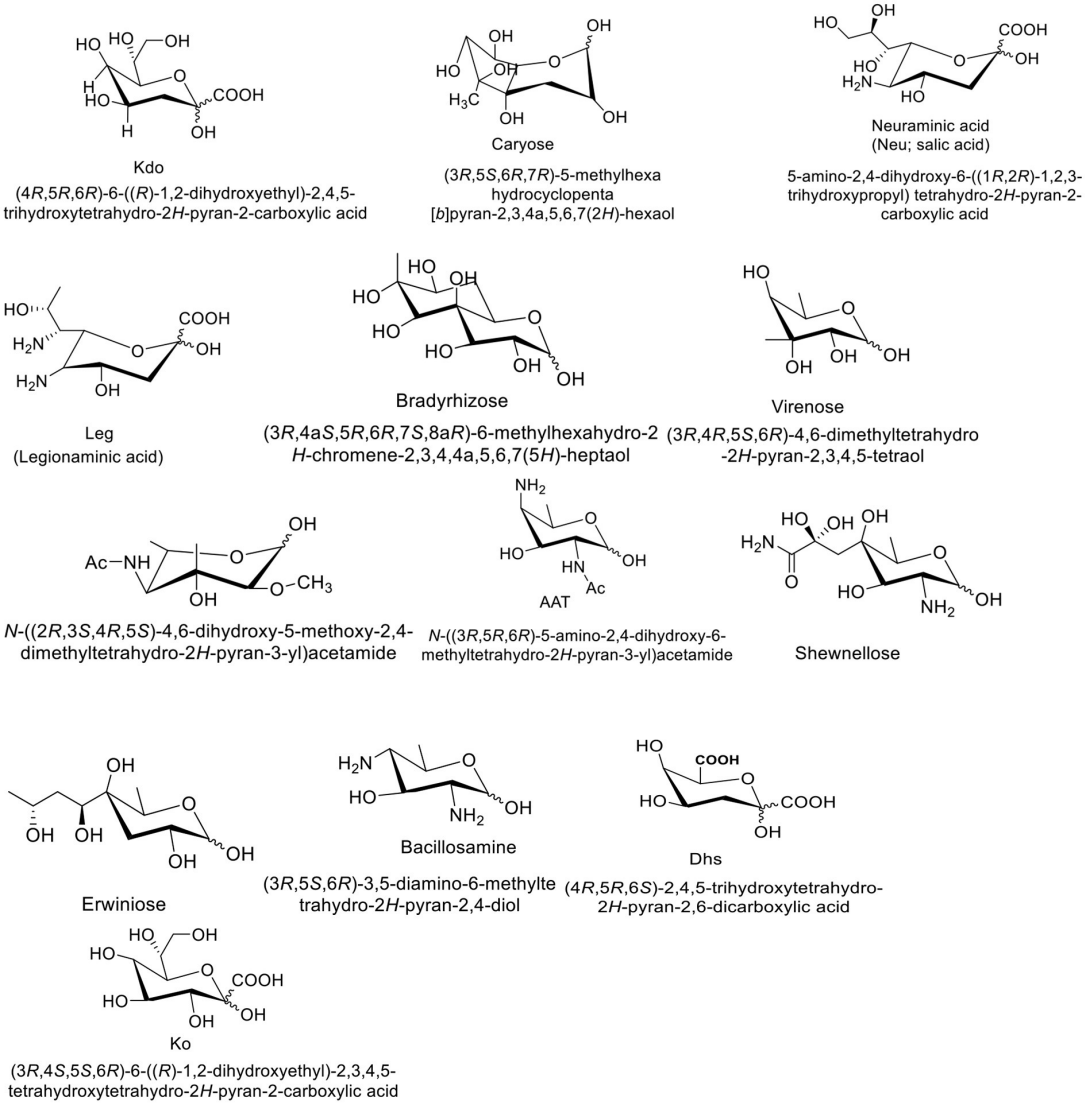
Structures of some unique bacterial monosaccharides.

Kdo (3-deoxy-d-manno-oct-2-ulosonic acid) is a type of monosaccharide that is generally found in Gram-negative bacteria (Figure 1). It helps in attaching surface polysaccharides to their lipid and function as a bridge between lipid A and the core oligosaccharide in lipopolysaccharides (16). Caryose (4,8-cyclo-3,9-dideoxy-l-erythro-d-ido-nonose) is another example of microbial monosaccharides which is a type of carbocyclic monosaccharide (Figure 1). It is produced by *Pseudomonas* species (17). Neuraminic acid (5-amino-3,5-dideoxy-D-glycero-D-galacto-non-2-ulosonic acid) is an acidic amino sugar with a backbone formed by nine carbon atoms. Some pathogenic bacteria obtain human N-Acetylneuraminic acid in the intestine and display on their own surfaces to attach Siglec-mediated host immunity (18). Another microbial monosaccharide is legionaminic acid, which is a nine-carbon diamino monosaccharide and found on the surface of bacterial pathogens. Due to its unique structure, it is considered an important biological probe (Figure 1) (19). Bradyrhizose is bicyclic monosaccharide which was first extracted from the *Bradyrhizobium* sp. (20). In gram-negative bacteria, some rare monosaccharides such as L-glycero-D-manno-heptose and D-glycero-D-manno-heptose along with Kdo are usually found in the inner core region of the lipopolysaccharides, and occasionally they are also present as a part of the outer core and O-antigens. As integral part of the LPS, these monosaccharides have significant role in host-bacterium interactions, therefore, they can be used for the inhibition studies of bacterial cell wall polysaccharide synthesis and stimulation of the host immune system (21).

Erwiniose is a 10-carbon branched monosaccharide unit which links to the α-D-Galactosyl residue of the backbone through (α1 → 3) glycosidic linkage (22). Bacillosamin is another characteristic bacterial monosaccharide which is found in the capsular polysaccharides and glycopeptides of various bacterial species. Chemically, bacillosamine is 2,4-diamino-2,4,6-trideoxy-hexose (DATDH) where N-acetyl groups are commonly attached to the C-2 and C-4 of the monosaccharide unit. In *Campylobacter jejuni* and *Neisseria gonorrhoeae*, DATDH is found at the reducing ends of both O- and N-linked glycoprotein-oligosaccharides (23). Similarly, D-glycero-D-talo-2-octulosonic acid (KO), is a high carbon sugar that is found in the lipoplysaccharide of gram-negative bacteria. The LPS is a part of the outer leaflet of bacterial cell wall, thus playing a crucial role in bacterial infections. Ko was isolated for the first time from the LPS component of *Acinetobacter calcoaceticus*. As compared to Kdo, the Ko glycosides contain C-3 hydroxyl groups, thus more resistant to acid hydrolysis. Several bacterial species such as *Acinetobacter hemolyticus, Yersinia pestis, Burkholderia cepacia complex,Serratia marcescens, and Acetobacter pasteurianus* show higher content of Ko in their lipopolysaccharides (24).

Bacterial polysaccharides can be used to create new biopolymers with unique characteristics. Other advantages of bacterial polysaccharides include the possibility of repeatable chemical and physical qualities, as well as a consistent supply of resources. A variety of potential bacterial polysaccharides can be developed commercially and used as a food additive. Polysaccharides are utilized as texture enhancers in food. In several countries including the European Union and the United States of America, the use of synthetic texture-promoting chemicals in food and dairy products is forbidden. Polysaccharides are useful for enhancing the appearance, stability, rheological qualities and nutraceutical values of food items (25). Based on their chemical composition, some of the common microbial polysaccharides are briefly discussed below.

### Alginate

Alginate is a linear and non-repeating exopolysaccharide and composed of β-D-mannuronic acid and α-L-guluronic acid. These subunits are attached through β-1,4-glycosidic linkages (26). Different bacteria belonging to the genera of *Azotobacter* and *Pseudomonas* produce alginates. Two uronic acid residues, compose the structure of alginate. They include β-D-mannuronic acid (M) and its C5 epimer α-L-guluronic acid (G), that are joined through 1,4-glycosidic linkages to each other. In nature, alginate varies considerably in its composition and length of M and G residues which can have a profound effect on the physiochemical characteristics of the polymer, as higher content of G residues is responsible for enhanced rigidity of the polymer (27). *Azotobacter vinelandii* is a bacterium that specifically produces alginate biopolymer. Alginate is associated to the wide category of linear chain polysaccharides. In the presence of certain cations such as Ca, K, Si, and Al, the polymer can form 3D networks. Alginate has applications in biomedical engineering, construction, food and feed industries, and agriculture sector (28).

### Bacterial cellulose

Several bacterial genera produce bacterial cellulose. Among them, *Salmonella, Acetobacter, Agrobacterium, Achromobacter, Aerobacter, Azotobacter, Sarcina ventriculi, Escherichia, and Rhizobium and Glucanacetobacter hansenii* based cell-free systems are some well-known examples (29). Bacterial cellulose is produced inside the bacterial cells in the form of β-1,4-glucan chains. These polymers are then excreted to the culture medium through the terminal complexes (TCs), which are found at the outside membrane of the cells. In the medium, these polymers crystallize and produce highly ordered structures such as protofibrils, ribbons, bundles which is finally converted to a hydrogel at the interface of air and medium (30). Bacterial cellulose performs several functions that are important for the life cycle of the organism. These functions include facilitation of plant attachment and flocculation. In comparison with plants' cellulose, bacterial cellulose possess many distinguishing features such as high purity, hydrophilic nature, and a finer three dimensional fibrous structure that are not found in plant-based cellulose (31).

### Hyaluronic acid

**Figure 2:**
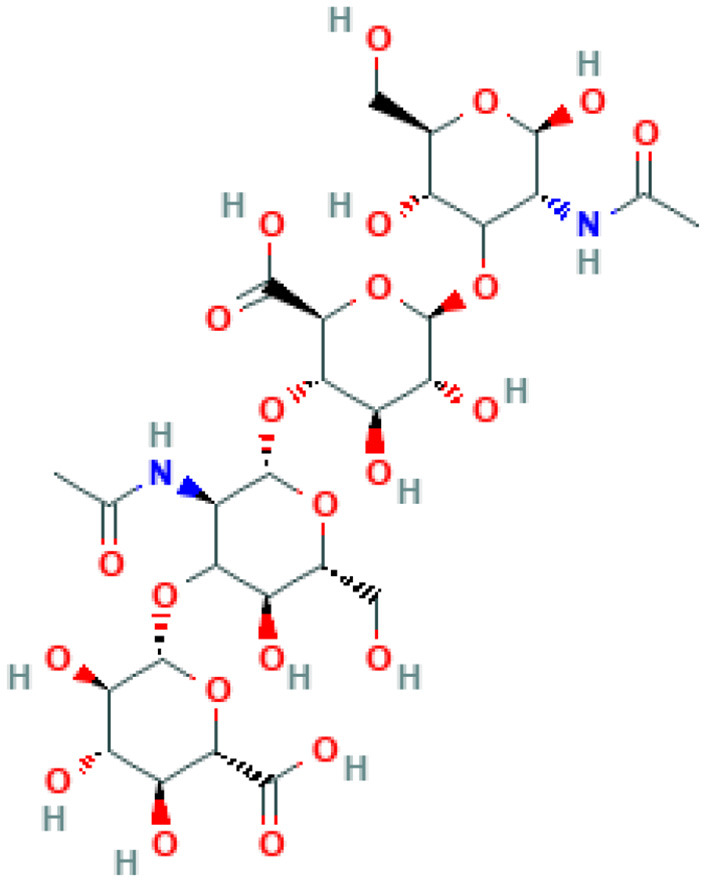
Structure of hyaluronan. The structure is obtained from PubChem NCBI.

Hyaluronic acid (Hyaluronan, C_28_H_44_N_2_O_23_) is a polymer of β-(1 → 4) linked D-glucuronic acid and N-acetyl-β-(1 → 3) linked D-glucosamine (Figure 2). Several bacterial species such as *Streptococci, Pasteurella multocida*, and *Cryptococcus neoformans* can produce hyaluronic acid. Scientists believe that the polymer helps microbes in immune evasion, and encapsulation of the bacterial cells, thus enabling them to escape the host's immune detection (32). Hyaluronic acid is also found in vertebrates. The extracellular matrix of vertebrate connective, neural, and epithelial tissues is rich with this polysaccharide. Hyaluronic acid has some extraordinary qualities such as enhanced viscoelasticity, easy degradability, and low immunogenic potential, making it more suitable for bioprinting (33). Due to these characteristics, since 1950s, the polymer has been used for biomedical purposes.

### Gellan

Gellan is a linear molecule which backbone structures comprised of [4)-α-L-Rhap- (1 → 3)-β-D-Glcp-(1 → 4)-β-D-GlcpA-(1 → 4)-β-D-Glcp-(1 → ]n repeats. The bacterium *Sphingomonas elodea* produces gellan gum in the form of an anionic extracellular polysaccharide. The polymer is constructed by three repeating monosaccharide units: α-L-rhamnose, β-D-glucose, and β-D-glucoronate. Besides other applications, gellan is also used as a stabilizing and gelling agent. In the biomedical industry, it has been used in ophthalmic treatments and sustained drug release formulations (34). The polymer has been investigated for the wound dressings but owing to soft texture and low thermal stability, the applications are not successful like other bacterial polymers such as bacterial cellulose. However, advancement in the field of 3D printing technology has overcome this issue up to some extent. Gellan gum has certain features that make it a suitable candidate to be in inks for bioprinting. Some of these properties include crosslinking potential mediated by very low cation concentrations, excellent gel formation capacity at 37°C, enhanced mono-dispersity, low immunogenicity, and outstanding rheological properties (35).

1n 1988, after a series of toxicological tests, Japan approved the use of gellan gum in food products and later by the USA (1990) and Europe (1995) (36, 37). The consumption of gellan has been reported with no adverse effects. Gellan is a food additive that can be utilized in a variety of ways in the food industry including jams and jellies, water-based gels, pie fillings and puddings, fabricated foods, icings and frostings and dairy products (ice cream, yogurt, milkshakes, and gelled milk). It has been used as a hydrocolloid coating for cheese (38). Gellan can also be used to substitute undesirable ingredients, such as fat, in order to create satiety-inducing products or low-calorie meals (39).

### Dextran

Dextran is an α (1 → 6) glucan with the side chains attached through α-(1 → 2)/α-(1 → 3)/α-(1 → 4) glycosidic linkages (40). Dextran is a natural polymer formed by some lactic acid producing bacteria such as *Leuconostoc mesenteroides* and *Streptococcus mutans*. Dextran is composed of glucopyranosyl residues linked through α-(1 → 6) and α-(1 → 4) linkages with each other. The polymer was discovered by Louis Pasteur for the first time as a by-product of wine fermentation. It became the first ever microbial polysaccharide to be applied in clinical settings and approved as plasma volume expander in the 1950s (41). The polymer is widely used as a major ingredient of hydrogels of burn wound dressings. As a part of the dressings, it promotes rapid formation of the new functional blood vessels and enhances the process of wound healing (42).

In the food industry, dextran is well-known for its viscosifying, emulsifying, texturizing, and stabilizing properties. Dextran has the potential to be used in place of commercial hydrocolloids as a new alternative in the food industry (43). For the separation and purification of the protein, cross-linked dextran, also known as Sephadex, is frequently employed. Dextran is now utilized in the culinary sector as a thickening for jam and ice cream. It prevents crystallization of sugar, improves moisture retention, and maintains the flavor and appearance of various food items (44, 45). It is frequently utilized as a carrier for medications, proteins, and other bioactive compounds as well as an antithrombotic agent. Dextran hydrogels were employed to create a photo-crosslinked tissue sticky gel that can hasten the healing of surgical wounds. The site-specific controlled distribution of several insoluble medications including ibuprofen, paclitaxel, and dexamethasone is another use for dextran hydrogels (46). It is a good nanocarrier for drug administration because of its low viscosity and gelation behavior in aqueous solutions. Cytotoxicity experiments revealed that the drug-loaded dextran nanocarrier indicated a substantial anti-cancer impact compared to the free drug (47). Dextran has been successfully used for targeted drug delivery of doxorubicin (DOX) for the treatment of cancer (48). In peripheral artery disease, dextran decreases *in vitro* platelet aggregation (49).

### Xanthan

Xanthan is an important member of exopolysaccharides (EPs), first isolated in 1959, and it is produced by a bacterium *Xanthomonas campestris, X. phaseoli and X. juglandis* through the process of aerobic fermentation of glucose and sucrose. Xanthan belongs to the sub-group of heteropolysaccharides, and is comprised of monomeric units of glucose, mannose, glucuronic acid, pyruvate, and acetate. The polymer has the intrinsic property to function as thickening agent, therefore used in food industry for the last 50 years. As xanthan is an inert polymer and has been used as a food additive. It has been recently focused for its application in the 3D printing technology. At low concentrations, xanthan has shear thinning capacity and viscosity, the desired characteristics due to which it can function as a rheological modifier, thus improving the 3D printing potential (50, 51). These characteristics of xanthan have also made it to be an essential part of some hydrogels employed for the purpose of tissue regeneration. Just like other inert bacterial polymers, the incorporation of some antimicrobial agents have enhanced the functional properties of xanthan, allowing it to be utilized for the targeted therapy of infections in various hydrogel formulations (52).

It is commonly utilized in the food sector. Food and Drug Administration (FDA) approved xanthan as a food ingredient in 1969 and Europe in 1974 as well (36). In the food industry, xanthan is used to make sauces, gravies, and sweets that may be chilled or cooked without losing their texture. It improves the storage of batter and dough, which is considered to enhance the elasticity of batters and doughs, and hence improve the retention of gas during the rising and proving stages (53).

### Curdlan

Curdlan is exopolysaccharides produced by bacteria. Curdlan is a linear water-insoluble glucan. Curdlan is a bacterial polysaccharide, first obtained in the year 1966 from the culture of *Alcaligenes* spp. (54). It can also be obtained from other bacterial species, including *Agrobacterium* spp., *Rhizobium* sp., *Bacillus* sp. and *Cellulomonas* sp. *Alcaligenes* spp., and *Paenibacillus* spp. (55). This type of polysaccharide is soluble in alkaline solution and insoluble in water. Curdlan is composed mainly of glucose units joined through linear β-(1, 3)-glycosidic linkages (56). Its name comes from the word “curdle,” which refers to its gelling tendency at high temperatures. The polysaccharide was initially identified in 1964, and its capabilities as a gelling agent were found to be peculiar and intriguing. Curdlan has the characteristic feature to form gel on heating, hence used as a food additive. The polymer is also used as a food additive as it enhances the stability, creaminess, viscosity, and elasticity of the food products. As curdlan is non-toxic and biodegradable in nature, therefore, the polymer and its derivatives are under investigations to be used as environment friendly alternatives to the non-degradable plastics derived from oil (57). Curdlan has been authorized as a food ingredient by the FDA. Curdlan has been employed in a wide range of food applications in Korea, Taiwan, and Japan. It also has a lot of bioactive properties (36, 58).

Curdlan and its derivatives have shown a wide range of pharmacotherapy functions, such as antiparasitic (inhibits the development of the malarial parasite *Plasmodium falciparum*.) anti-tumor (blocks the immune suppression by myeloid-derived suppressor cells and reduces tumor burden), antiviral (inhibits the development of dengue and hepatitis B virus infection), antifungal (particularly, against *Aspergillus fumigatus*), immunomodulation (stimulates CD4^+^ and CD8^+^ T cells), anti-coagulation. Due to their unique immune-regulatory properties, curdlan and its derivatives have the potentials to be employed as vaccine adjuvants that could boost vaccination immune responses (54). Furthermore, because curdlan is not easily hydrolyzed by digestive enzymes, it can be used to supplement prebiotics, minimize calories, and support healthy gut flora, all of which help to prevent obesity (55). In another study, Curdlan-graft-poly(ethylene glycol) has been employed to administer the chemotherapy medication doxorubicin (59).

Based on cellular location, microbial polysaccharides have been divided into the following categories.

## Microbial polysaccharides based on cellular location

### Intracellular polysaccharides

Majority of the intracellular polysaccharides (IPS) are found in fungi such as *Pleurotus* sp., *Ganoderma* sp., *Phlebia* sp., *Inonotus* sp., *Tricholoma mongolicum* and *Trametes versicolor* whereas some bacteria such as *Bacteroides* also produce them (60, 61). Other bacterial species that produce intracellular polysaccharides include *Xanthomonas campestris, Bacillus mojavensis*, and *Lactobacillus reuteri* (62). Several intracellular polysaccharides have exhibited excellent biological activities such as antioxidant, anti-tumor as well as immunomodulatory activities, thus making them good candidates for biomedical and pharmacological applications (63). Zhang et al. (64) studied *Pleurotus eryngii* (SI-04) for the production of intracellular polysaccharides, and it was found that two types polysaccharides, i.e., IPS-1 and IPS-2 were produced. Further these polysaccharides were investigated for their hepatoprotective and antioxidant activities. The IPS-2 displayed superior hepatoprotective ability by lowering concentrations of triglycerides and albumin in the serum. On the other hand, serum bilirubin level was found to be increased, thus the antioxidant status of the liver was improved. In another study conducted by Hao et al. (65), intracellular and exopolysaccharides obtained from *Fomitopsis pinicola* were studied their functional characteristics. Both types of the polysaccharides exhibited excellent antioxidant properties, thus showed their potential to be used as suitable candidates in the field of industrial biotechnology and biomedical engineering.

### Structural polysaccharides

A variety of polysaccharides such as cellulose, starch, chitin, and peptidoglycans, are involved in maintaining structural integrity of the microorganisms, hence they are known as structural polysaccharides. These polysaccharides provide shape and firmness to the microbial cells. Using both physical and chemical methods, Frei and Preston (66) investigated the cell wall structures of various algal species such as Caulerpa, Udotea, Halimeda, Penicillus, and Dichotomosiphon. Their study revealed the absence of cellulose from the cell wall and the main structural component was found to be a polymer named as β-1,3-xylan. The cell wall of algae contains sulfated polysaccharides, where they perform significant biological functions. These polysaccharides also display some outstanding biological and pharmacological characteristics such as antioxidant and anti-inflammatory activities. Although, some other important biological properties including antimicrobial, anti-tumor, immunomodulatory of these structural polysaccharides have been investigated and are widely reported in the literature (67).

### Extracellular polysaccharides

Microbes produce a variety of extracellular polysaccharides. The extracellular polysaccharides have been classified into four major categories, and include amicrocapsular and slime polysaccharides, polyamides, inorganic polyanhydrides, and polyesters. These polysaccharides are collectively called as exopolysaccharides (EPSs) (62). Sutherland (68) introduced the term “exopolysaccharides” for the first time for high molecular weight polymeric carbohydrates that are produced by various microorganisms. The category of EPSs is primarily comprised of carbohydrates having different structures and compositions. Based on their monosaccharide components, EPSs have two types (a) homopolysaccharides; composed of single type of monosaccharide units linked through glycosidic bonds, and (b) heteropolysaccharides; contain more than one type of monosaccharide units. Harsh environments are thought to be important sources for industrially important microbes. These microorganisms are the producers of a variety of active biological molecules, such as polysaccharides, glycoproteins, and proteins, among others. Exopolysaccharides, especially from extremophilic bacteria, have attracted attention of the researchers for their potential to be used for nanoencapsulation of anti-cancer drugs and the ability of sustained release and antineoplastic properties. Halophilic bacteria can live under high salt stress, ranging from 5 to 25% of sodium chloride. High molecular weight polysaccharides (EPs) with potential biological activities were obtained from *Halomonas* species, and the monosaccharide units were found to be glucose, galactose, mannose, and glucuronic acid. As the polysaccharide was rich with uronic acid and sulfate moieties, so it showed high anionic properties. Some outstanding characteristics of the polysaccharide such as viscoelasticity, pseudo-plasticity, and thixotropic nature prove them a good candidate to be employed for the fabrication of modern material. Similarly, other factors such change in pH, the presence of any surfactant, salts, sugars, lactic acid, and alternate freeze thawing had no effect on properties (69). The polysaccharides also showed the potential to bear and stay strong under various environmental insults such as extreme pH, elevated temperature, freeze thawing, and high salt concentrations. The biopolymers can also help in cleaning contaminated water by binding various heavy metal ions and produce stable gels. Further, the rich sulfate nature of the EPSs may be responsible their anti-cancer and immunomodulatory potential against malignant cells (70).

## Prebiotic properties of microbial polysaccharides

### Anti-inflammatory effects

A substantial literature has been generated on the anti-inflammatory effects of polysaccharides obtained from terrestrial and marine resources (71–75). Albeit the studies regarding the anti-inflammatory effects of bacterial-derived polysaccharides are limited. Although, few bacterial-derived polysaccharides have been reported for inducing anti-inflammatory effects in Inflammatory Bowel Disease (IBD) patients (72), however, extensive research needs to be carried out to discover and define more bacterial polysaccharides. Some polysaccharides (particularly, those produced by *Helicobacter hepaticus)* are capable of triggering an early induction of IL-10 in the intestine, which in turn can activate the MSK/CREB-dependent anti-inflammatory response and repair gene signature *via* the Toll-like receptor 2 (TLR2) (76). Such interaction between host and bacteria at the intestinal interface is of mutualistic nature and can potentially be utilized in designing novel future strategies to tackle various gut inflammatory disorders with a particular emphasis on IBD.

Zwitterionic capsular polysaccharides (ZPSs), produced by bacteria, are known to modulate anti-inflammatory IL-10-secreting T regulatory cells (74, 77). When human mononuclear cells were exposed to lysates of these putative ZPS-producing bacteria, a resultantly higher IL-10 production was noted compared to control. ZPSs activate the adaptive immunity of a host *via* antigen-presenting cells (APCs), which is then followed by the presentation of these ZPSs, by using the major histocompatibility complex class II pathway, to CD4(+) T cells (78). This discovery was the first mechanistic insight into how carbohydrates can activate T cells. Through their ability to activate CD4(+) T cells, ZPSs direct the cellular and physical maturation of the developing immune system. It is now evident that T cell activation by ZPS requires prior interactions of T cells with APCs (79). Thus, bacterial ZPS are directing the maturation of the developing immune systems *via* activating CD4+ T cells. Bacteria like *Bacteroides fragilis* are known to produce ZPS (74). A genomic screen study has identified a diverse group of host associated bacteria that produce ZPSs (74). Such ZPS-producing bacteria could potentially be used as probiotics, particularly in the treatment of gut inflammatory disorders including IBD.

**Figure 3:**
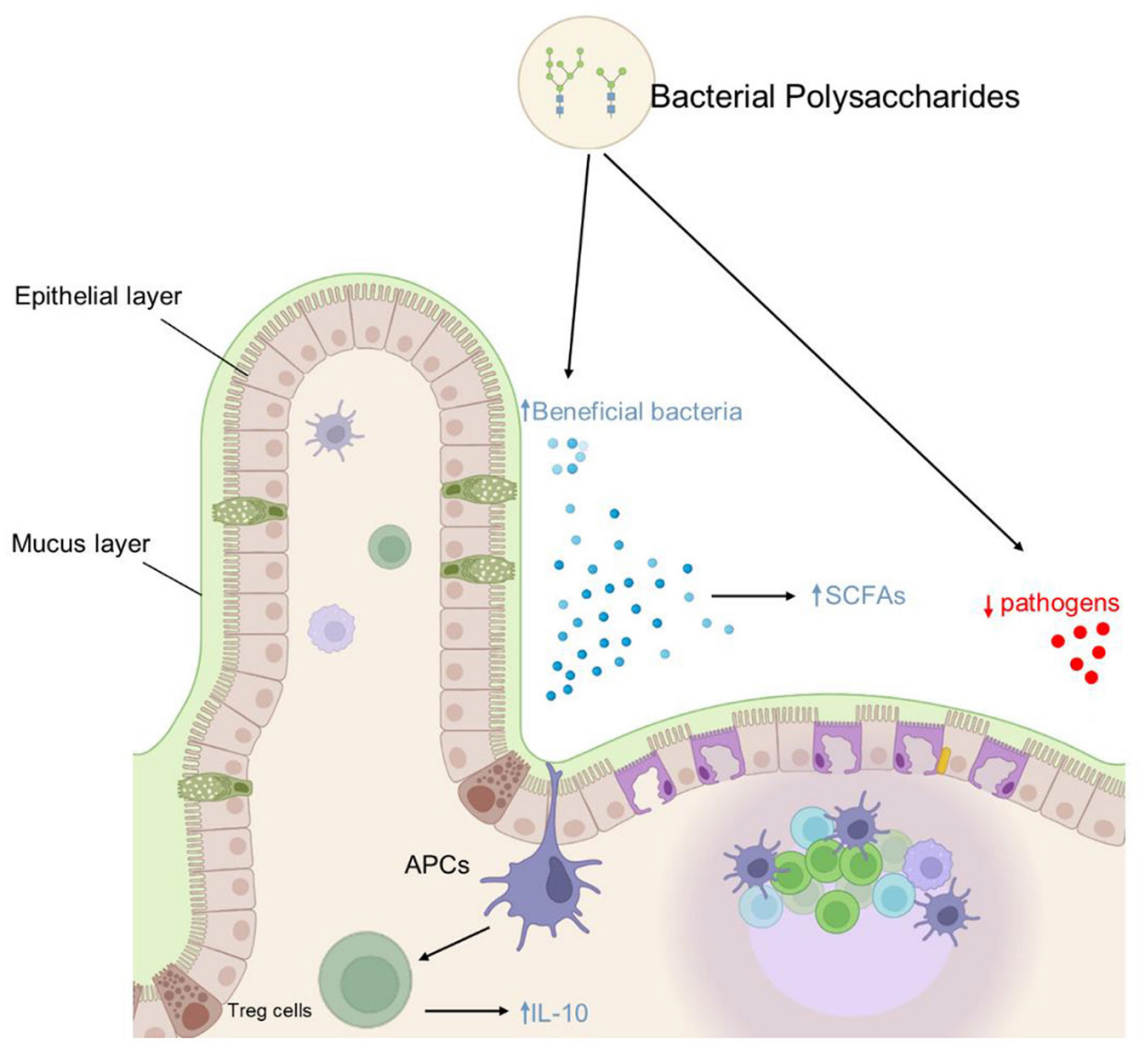
Schematic diagram of anti-inflammatory and GM modulation effects of bacterial polysaccharides: polysaccharides have anti-inflammatory properties that are mediated by a variety of mechanisms, including early IL-10 induction, increased CD3+ T cell population, increased SCFA producing bacteria, and decreased potential pathogen population in the gut.

Capsular polysaccharides of serotypes 5 and 8 of *Staphylococcus aureus* (a clinical isolate) are known to have a significant role in the virulence and pathogenicity of this pathogen (80). Purified forms of these serotypes capsular polysaccharides could potentially be utilized in the development of vaccines against staphylococcal infections. Similarly, the EPSS from *Leuconostoc mesenteroides* species are potential immunostimulants and capable of inducing IgA in the intestine (81). Further, *In vitro* analysis of this EPSs revealed that it can modulate Th1 and Th2 cell-mediated responses in splenocytes. This EPSs, when orally administered, induced fecal IgA production, up-regulated the retinoic acid synthase and growth factor-β receptor genes, and increased the CD3+ T-cell population. This further suggests that *Leuconostoc mesenteroides* derived EPSs has an immunomodulatory effect thereby enhancing the mucosal barrier. Contrastingly, EPSs from *Lactobacillus rhamnosus*, was revealed to have pro-inflammatory effects (82). Similarly, the well-known *Bifidobacterium spp*. harbor genes encoding for EPSs production and have a role in the immunomodulation of their host (83). Furthermore, it is important to look for the lower molecular weight polysaccharides of a heavy EPSs produce by microorganism. For instance, *Lactobacillus confusus* TISTR 1498 produced EPSs were incompetent to exert and immunomodulatory activity. However, the lower molecular weight partially hydrolized EPSs (M_w_ values ≤ 70 × 103 g/mol) stimulated RAW264.7 cells to produce pro-inflammatory mediator nitric oxide (NO) and cytokines (84). Therefore, we propose that extensive work be carried out to discover potent bacterial polysaccharides and define their function and possible application for the welfare of humanity. Moreover, it would be interesting to identify the fraction of the GM involved in various polysaccharide synthesis or polysaccharide modulation and consequently define their effects on the host immune system and overall health. Also, emphasizing on the bacterial derived polysaccharides mediated immunomodulation, their effects need to be evaluated in providing protection to host against various pathogenic microorganisms. Various anti-inflammatory potentials of bacterial polysaccharides are summarized in Figure 3.

### Antioxidant properties

Antioxidants are low molecular weight compounds that scavenge free radicals including reactive (hypochlorite) and non-reactive species (reactive oxygen and nitrogen species) (85). Free radicals can be formed because of the natural environment, external stimuli, and the *in-vivo* metabolism of compounds. Free radicals are associated with human diseases, including Alzheimer's disease, atherosclerosis, Parkinson's disease, cancer, and many others. They can be found in food, medicines, drinking water and the air we breathe (including fried foods, alcohol, cigarettes, air pollutants, pesticides, UV). Additionally, free radicals are byproducts of chemical reactions, such as metabolism (85, 86). The creation and removal of free radicals in the human body are in a dynamic equilibrium and any disturbances to this balance could result in the accumulation of free radicals that could trigger a range of disorders such as cancer (87), diabetes (88), cardiovascular disease (89), and Alzheimer's disease (90). Thus, the development of antioxidant medications may efficiently reduce the overproduction of free radicals and prevent and treat disease.

Polysaccharides with biological function are known as “biological response modifiers” (91). Bacterial polysaccharides demonstrate antioxidant potential against the free radicals or reactive oxygen species (ROS) due to oxidative burst. In a study, *in vitro* antioxidant activity has been reported for a polysaccharide derived from the broth of cultivated *Streptomyces virginia* H03 (92). The antioxidant polysaccharides obtained from *Streptomyces virginia* showed better stability to heat compared to vitamin C. The antioxidant properties of the polysaccharide remained nearly unchanged after being heated from 50 to 100°C for 5 min, whereas the antioxidant properties of vitamin C decreased dramatically from 50 to 100°C. Similarly, *Aerococcus uriaeequi* produced polysaccharides have shown excellent results against hydroxyl radicals (•OH) in a dose-dependent manner. When *A. uriaeequi* polysaccharide concentration reached 100 μg/mL, the inhibition rate reached 45.65%, which is lower than the value of Vitamin C (standard). Whereas, *A. uriaeequi* polysaccharides' activity against superoxide anion radical (O_2_·^**−**^**)** reached 250 g/mL with an inhibition rate of 67.31%, which is close to Vitamin C. The findings suggest that the polysaccharides of *A. uriaeequi* may be applied as a novel potential antioxidant (93). Polysaccharides obtained from the culture of *Enterobacter* species have also shown antioxidant abilities. These polysaccharides have shown significantly higher antioxidant activities against superoxide (IC_50_ 0.33 mg/mL) and 2,2-diphenyl-1-picryl-hydrazyl-hydrate (DPPH) (IC_50_ 0.44 mg/mL) (94). Bacterial polysaccharides obtained from *Bacillus amyloliquefaciens* indicated antioxidant properties with IC_50_ = 0.21 μg/mL values for DPPH, 30.04 μg/mL for H_2_O_2_ and 35.28 μg/mL for superoxide. Bacterial polysaccharides included uronic acid (12.3%) and sulfate (22.8%), with a molar ratio of 1:1:9 for glucose, galactose, and glucuronic acid, respectively (95).

Polysaccharides producing bacterium *Bacillus thuringiensis* showed potent antioxidant properties in an investigation against DPPH (79% at 1.0 mg/mL) and superoxide (75.12% at 1.0 mg/mL). *Bacillus thuringiensis polysaccharides were comprised of* fructose (43.8%), galactose (20%), xylose (17.8%), glucose (7.2%), rhamnose (7.1) and mannose (4.1%) (96). The polysaccharide obtained from *Halolactibacillus miurensis* indicated its potential antioxidant activities against all free radicals (97).

Bacterium *Polaribacter* sp. SM1127 produced polysaccharides have a molecular mass of 220 kDa and are mostly composed of N-acetyl glucosamine, mannose, and glucuronic acid residues linked by heterogeneous linkages. The antioxidant activity of the polysaccharide obtained from *Polaribacter* sp. SM1127 efficiently scavanged free radicals at a concentration of 10.0 mg/mL. The scavenging ratios for DPPH•, •OH, and O_2_ – • of the polysaccharide were 55.4%, 52.1% and 28.2%, respectively (98).

Another polysaccharide has been reported with antioxidant characteristics which is produced by the bacterium *Haloterrigena turkmenica*. The bacteria mostly excrete the polysaccharide in the middle exponential growth phase and reach their maximal production in the stationary phase. Anion exchange chromatography and SEC-TDA Viscotek analyses revealed that the polysaccharide was made up of two major fractions that were 801.7 and 206.0 kDa in size. It was a sulfated heteropolysaccharide containing glucose, galactose, glucuronic acid, glucosamine, and galactosamine. The polysaccharide showed potent antioxidant properties in an investigation against DPPH (68.2% at 10 mg/mL) (99). In summary bacterial derived polysaccharides can be utilized as potential alternatives to conventionally used antioxidants such as vitamin C. Similarly, they can be utilized as a potential therapeutics and also in various biotechnological applications.

### Anti-cancer effects

Due to the potent therapeutic abilities of bacterial polysaccharides, interests are developing to harness the potential of bacterial polysaccharides for cancer treatment. Polysaccharides possessing sulfur and uronic acids displayed antioxidant potential and anti-cancer properties by restoring cell redox control (100, 101). The most prevalent mechanisms of anti-tumor polysaccharides are cell cycle arrest, anti-angiogenesis, and apoptosis, all of which have direct tumor-killing abilities (102). Polysaccharide anti-tumor action is determined by the main chain configuration (103).

It is known that microbial exopolysaccharides trigger the immune system *via* MAPK and NF-κB pathways (7). Anti-tumor activities of *B. amyloliquefaciens* against human breast adenocarcinoma (MCF7) and human prostate cancer (PC3) have been investigated, as well as the suppressive effect of bacterial polysaccharides on Ehrlich ascites carcinoma. Polysaccharides indicated a powerful and selective impact on MCF7 breast cancer cells, with a death percentage of 65.20% with IC_50_ = 70 μg/mL and IC_90_ = 127.40 μg/mL. These polysaccharides reduced the number of viable EAC cells and induced non-viable cells (95). *Bacillus thuringiensis* produced polysaccharides have shown potent anti-cancer abilities when tested against the Lung cancer cell (A549), Liver cancer cell (HepG-2) and Normal fibroblast cell (Vero cell lines). Interestingly, these polysaccharides showed potential cytotoxicity against the cancer cell lines A549 and HEp-2 compared to normal Vero cells (96).

The anti-tumor activity of *Rhizobium* sp. polysaccharide (at a concentration of 5–120 mg/kg) has been studied in mice with sarcoma 180 (S180), hepatoma 22 (H22), and Ehrlich ascites carcinoma (EAC), respectively. The high inhibitory rate was 44.17% at a concentration of 10 mg/kg against S180, 55.80% at a concentration of 10 mg/kg against H22, and 53.10% at a concentration of 60 mg/kg against EAC. Apparently, no side effects of the polysaccharides were noticed in the mice in terms of body weight (104).

*Pseudomonas* spp. WAK-1derived polysaccharides that have a repeating unit:−2)-β-D-Galp(4SO4)(1–4)[β-D-Glcp (1–6)]-β-D-Galp(3SO4)(1- can effectively inhibit human cancer cell lines such as central nervous system cancer and lung cancer cell lines. The average polysaccharide dosage needed for 50% growth inhibition against the cell lines was 63.2 μg/mL. High sensitivities to polysaccharide were noticed in central nervous system cancer and lung cancer cell lines among the cancer cell lines evaluated (105).

Polysaccharide isolated from *Lactobacillus acidophilus* has suppressed colorectal cancer cells (HT-29) in *in vitro*. These polysaccharides markedly remodeled the cell shape and surprisingly no significant changes were noticed in the nucleus condensation and cell cycle (including the G_0_-G_1_ and S phases) (106).

Polysaccharides derived from marine bacterial species, *Bacillus megaterium*, showed cytotoxicity against Hepatocellular carcinoma (HepG2) cells with IC_50_ value of 218 μg/mL. The presence of sulfur and uronic acids in the polysaccharide structure contributed to the high cytotoxicity (100). Polysaccharides marinactan, derived from the marine microorganisms *Flavobacterium uliginosum*, completely eradicated a sarcoma-180 solid tumor in all the mice treated for 10 days. The polysaccharide consists of glucose, mannose, and fucose with a ratio of 7:2:1. The exposure to bacterial polysaccharide significantly increased the survival time of mice with ascites sarcoma 180. Marinactan has minimal acute toxicity; mice were not dead when given 100 mg/kg intraperitoneally or subcutaneously (107).

Since polysaccharides are biocompatible, biodegradable, non-toxic, and non-immunogenic, they can also serve as efficient drug delivery systems for a variety of drugs, including anti-cancer drugs (108). An effective candidate for a drug delivery system is alginate, obtained from bacteria and seaweed. Different anti-cancer drugs, such as Curcumin diglutaric acid (109), Doxorubicin (110), Curcumin and 6-gingerol (110), Cisplatin Temozolomide and doxorubicin (111), are transferred using an alginate-based drug carrier. Dextran is another natural polysaccharide, produced by Leuconostoc spp. and Lactobacillus spp. Dextran and its derivatives are suitable nanomaterials for drug delivery and have gained great attention in the development of drug delivery systems. The dextran-based delivery systems can boost intestinal epithelial absorption and protect drug molecules from enzymatic and chemical destruction across the stomach and small intestine, enhancing oral bioavailability (108). Anti-cancer drugs including Doxorubicin (DOX), silybin, paclitaxel (112), DOX, bortezomib (113), Rapamycin (114), siRNA/paclitaxel (115), Asiatic acid (116) are transferred using a dextran-based delivery system.

Gellan is produced by *Sphingomonas paucimobilis*. A new gellan gum nano hydrogel system that can transport and administer anti-cancer drugs has been reported. The Gellan gum nano hydrogel system helps in the transfer of multidrugs, including prednisolone and paclitaxel, which could be very useful for the treatment of inflammatory carcinoma or several types of prostate cancer. Prednisolone can act on cancer cells to limit their biological proliferation and lessen the negative effects of chemotherapy. By combining this impact with paclitaxel's toxic properties, one can have a synergistic and more toxic effect on several cancer cell types (117). From the above data, polysaccharides can be useful for biotechnological and pharmaceutical applications as they showed cytotoxic and anti-cancer activities.

### Antibacterial, anti-fungal, and antiviral properties of bacterial polysaccharides

Naturally occurring polysaccharides are known for their antimicrobial potential against a variety of bacterial pathogens by interfering with their biofilm formation, quorum sensing, efflux pump, bacterial cell wall/cell membrane synthesis, and bacterial nucleic acid synthesis (118). Interestingly, some bacteria can also produce polysaccharides that exhibit antimicrobial activities against specific pathogens. For instance, *Lactobacillus plantarum* and *Bacillus* spp. secreted polysaccharides can inhibit the efflux pump of *Escherichia coli* ATCC35218 biofilm and thereby leave the cells susceptible to low-grade antimicrobial agents (119). Similarly, the EPSs sourced from probiotic bacteria (e.g., *Lactobacillus acidophilus* A4) was capable to repress the expression of certain genes including (*crl, csgA*, and *csgB*, and *cheY*), thereby resulted in the inhibition of antibacterial resistance in *E. coli* O157:H7 (120). Moreover, bacterial EPSs can also affect the permeability of a bacterial pathogen's cell wall. For instance, the EPSs secreted by *Streptomyces virginia* H03 strain lead to increased permeability of the cell wall of *Staphylococcus aureus* ultimately resulting in cell leakage (121). In addition, EPSs from *Lactobacillus plantarum* and *Bacillus* spp. are known to reduce antimicrobial resistance in *Escherichia coli* ATCC35218, by affecting their cell membrane function (119). Also, *Bifidobacterium longum* BCRC 14634 strain-derived EPSs showed inhibitory activity against seven bacteria involved in food spoilage and infection (122).

The antimicrobial activity of bacterial-derived polysaccharides can be classified into antibacterial, antifungal, and antiviral polysaccharides. EPSs from a variety of bacterial strains belonging to the genera *Lactobacillus, Lactococcus* and *Streptococcus* are known to have antibacterial activities against a variety of pathogenic bacteria (123). These EPSs showed strong antibacterial activities against both gram-positive and gram-negative pathogenic bacteria such as *Enterococcus faecalis, Listeria monocytogenes, Staphylococcus aureus, E. coli, Pseudomonas aeruginosa, Salmonella typhimurium*, and *Shigella flexneri* (123). Moreover, antibiofilm activities of various *Lactobacillus* and *Enterococcus* genera derived polysaccharides have been documented against a variety of both gram-positive and gram-negative pathogenic bacteria. These polysaccharides affected biofilm formation in *E. Faecalis, L. monocytogenes, S. aureus, P. aeruginosa, S. typhimurium, E. coli* and others (123). For instance, EPSs isolated from *Enterococcus faecium* MC13 inhibited biofilm formation in *L. monocytogenes* (124). Additionally, an EPSs producing *Lactobacillus casei* probiotic strain when co-cultured with various pathogenic bacteria, inhibited the growth of *B. cereus, S. aureus, S. typhimurium* and *E. coli* O157:H7 (125).

**Figure 4:**
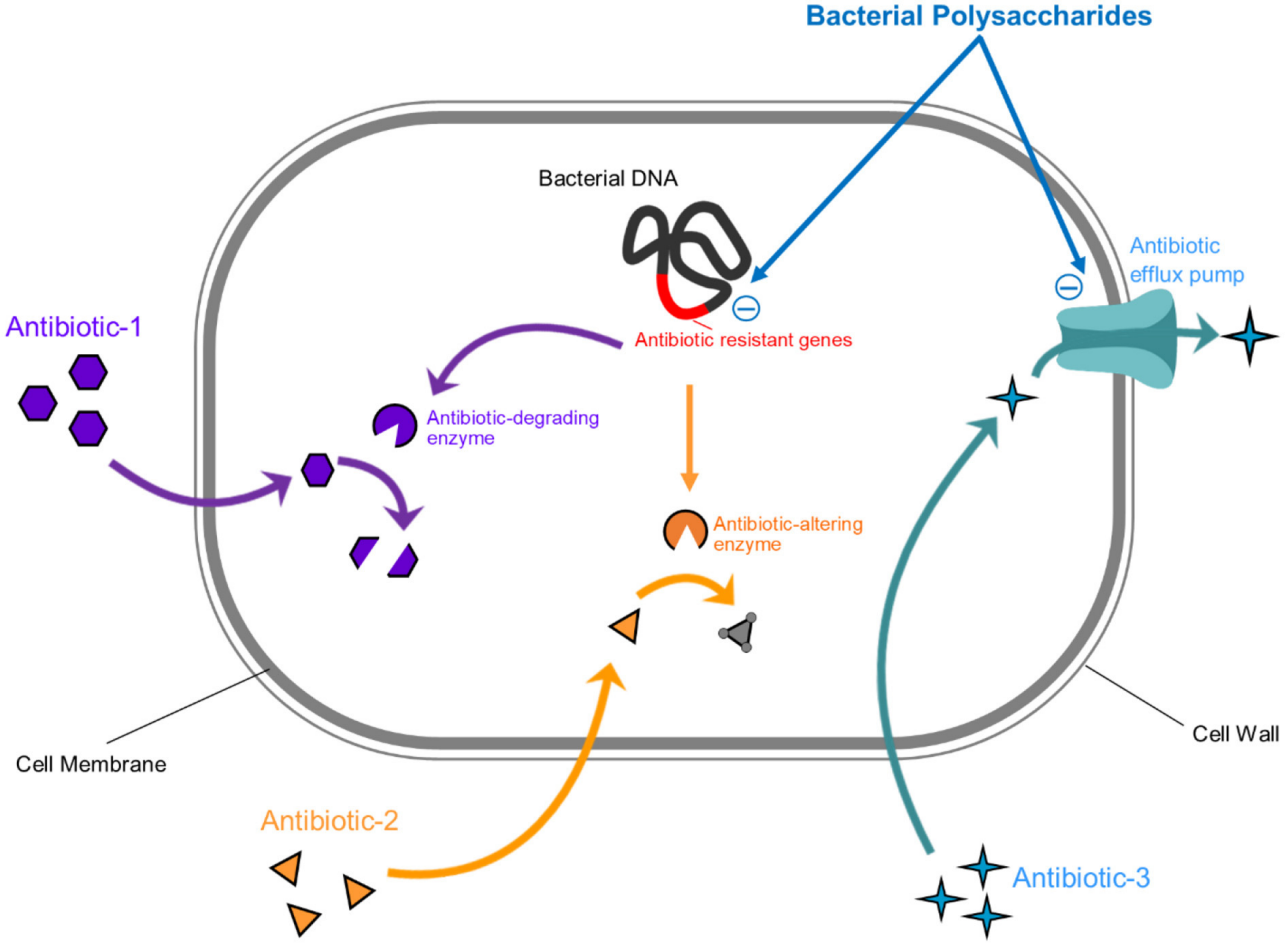
Graphical illustration of the antibacterial properties of bacterial-derived polysaccharides. Bacterial EPSs inhibit bacterial pathogens by (1) inhibiting efflux pump (which is responsible for removal of antimicrobial agent) and (2) inhibiting the expression of antimicrobial resistant genes.

The antifungal activity of bacterial derived polysaccharides has also been documented against various candida strains. For instance, polysaccharides from *Lactobacillus lactis* have a fungicidal effect against *C. albicans* (126). Similarly, the *Lactobacillus rhamnosus GG* strain produced polysaccharides which rendered the growth of *Candida albicans* and *Candida glabrata* by decreasing their hyphal formation (127). Moreover, various bacterial EPSs revealed antiviral activities against pathogenic viruses as well. These EPSs have potent antiviral properties and interfer with the life cycle of viruses or potentiate the host immunity against a viral infection (6). For instance, the Adenovirus type 5 growth was found to be affected in the presence of EPSs derived from *Lactobacillus* sp. which obstructed the HAdV-5 reproduction process (128). Bacterial EPSs mediated antiviral activities against other pathogenic viruses include those against rotaviruses, influenza viruses and Transmissible Gastroenteritis Virus (129–132). EPSs affect viral growth *via* various mechanisms including (i) interfering with the viral attachment to cells, (ii) inducing the expression of IFN-α, IFN-β, MxA and RNase L, and (iii) reducing viral replication rate. Therefore, these bacterial EPSs can be utilized as unique agents for targeting human pathogens. Although the studies regarding the antimicrobial effects of bacterial secreted polysaccharides are limited, still considering their huge potential, there is a need to explore novel EPSs from the large diversity of bacteria and to assess their potential against various pathogenic microorganisms. This could be a potentially sustainable solution to the growing concern of antimicrobial discovery and to combating antimicrobial resistance. Moreover, bacterial polysaccharides based postbiotics combinations with antimicrobial properties could be better candidates in tackling polymicrobial infections. Still, studies regarding the safety and resistance related concerns of these antimicrobial polysaccharides need to be conducted. Figure 4 summarizes the mechanisms of bacterial EPSs through which they can affect a bacterial pathogen.

### Gut microbiota improving properties

Polysaccharides of microbial origin have been known not only to affect the GM structure and function, but have shown an overall improvement in gut health (75). Bacterial EPSs can modulate the host-bacterial interactions directly through the adhesion of bacteria to gut epithelium, modulation of host immunity, and the function of GM (133, 134). EPSs secreted by certain gut bacteria can further be metabolized by some other commensal microbes (Figure 1). For instance, *Weissiella* and *Pediococcus* secreted EPSs can promote the growth of health-promoting members of the gut community including species from the genus *Bifidobacterium* (135). Another study investigated the role of a *Lactobacillus plantarum* NCU116 strain secreting EPSs (EPS116) in promoting intestinal epithelial regeneration. The EPS116 was found to promote intestinal homeostasis by modulating the intestinal stem cells' proliferation and differentiation, and by altering the structure of GM (136). In another study, the EPSs produced by two *L. paracasei* strains were metabolized by other GM members which resulted in a significant increase in the production of short-chain fatty acids (SCFA) (137). Moreover, the EPSs secreted by *L. reuteri* and *Bifidobacterium* are often degraded by *Bacteroides*, which further potentiate the SCFAs production in the gut and resultantly improve host health (138). It is also reported that various bacterial EPSs modulate the structure and function of GM that concurrently lead to improved health and in some cases have alleviated disease symptoms (139–141). EPSs producing strains can be utilized for targeted modulation of the GM, such as affecting the abundance of specific gut taxa (for instance, SCFA producers). Few metabolic conditions that can particularly be targeted with such interventional strategy include metabolism associated disorders, such as obesity, hyperlipidemia, type 2 diabetes mellitus (T2DM), non-alcoholic fatty liver disease (NAFLD), and metabolic syndrome (MetS). These conditions are usually induced by high-fat diet (HFD) intake and bacterial polysaccharide mediated interventions can be proposed targeting the core microbiota highly associated with these conditions. In summary, the polysaccharides secreted by bacteria can potentially be utilized as an interventive strategy to remodel the dysbiotic GM composition and fix metabolic and immune anomalies of the host.

### Postbiotics polysaccharides and potential future clinical implications

To modulate the gut dysbiosis, the utilization of prebiotics, probiotics, and postbiotics has emerged as an area of interest recently. Postbiotics is defined as any substance which is produced by or released *via* the microbial metabolism, and which can directly or indirectly exerts a beneficial effect on the host (142). Compared to probiotics there are minimal pathogenicity related risks associated with probiotics, because it does not contain any live microorganisms. The use of Postbiotics has been associated to have various effects on host including immunomodulatory, antioxidant, anti-inflammatory, and anti-cancer (142). Postbiotics have been classified into various categories including Cell-Free Supernatants, Enzymes, EPS, Cell Wall Fragments, SCFAs, bacterial lysates and metabolites produced by GM. Focusing on the bacterial EPS postbiotics, though their biological function is not entirely clear, still its potential use is of great interest in pharmaceutical products and functional foods to promote human health. For instance, the use of EPS has been known in immune modulation (143), enhancing phagocytosis in macrophages (144), and stimulated proliferation of lymphocytes (144) and have antioxidant and cholesterol lowering properties (145, 146). Therefore, these postbiotics can potentially be used in preventing cardiovascular diseases. Moreover, bacterial derived EPS has also been used as adjuvant which enhanced the efficacy of the foot-and-mouth disease vaccine (147). Some postbiotics such as β-glucans can activate macrophages and may promote cellular immune response against various pathogenic microorganisms including bacteria, viruses, parasites, and cancer cells (148, 149). Additionally, since β-glucans facilitates the adhesion of probiotics to the intestinal epithelium these can be used synergistically for a more pronounced effect of probiotics (28). There are variety of postbiotics, however, considering the current work done on the effect of probiotics to alter host microbiome, still there is a need of further research to assess the detailed safety and efficacy of Postbiotics.

### Bacterial polysaccharides in food and nutrition

Because of their special physiochemical characteristics, the use of microbial polysaccharides has gained importance as their utilization as food components in the food industry. A variety of bacterial taxa, produce levan, a microbial polysaccharide, which is found in the cytoplasm of these bacteria (150). It is used as a culinary additive as a flavor enhancer, as a prebiotic in yogurt, and to improve the texture of foods like bread, milk, drinks, and even cereal (151). Polysaccharide from microbial sources other than bacteria such as fungi are also of much importance in food industry and enhancing nutritional value of the food. For instance, pullulan is utilized in drinks as a low viscosity filter. Additionally, it serves as a binding agent in dishes like sauces and soups (152). Gellan another fungal polysaccharide, due to its propensity to create gel films it is frequently employed in the food business. Chitin and chitosan are abundant in the cell walls of filamentous fungus (153). The types of fungus most frequently utilized for producing chitin and chitosan for use as stabilizers in food. Polysaccharides made by fungi are of tremendous interest because of the wide range of uses they have in the food industry and as food. Fungi are a fantastic source of polysaccharides, which have several health advantages. The food industry uses hydrocolloids including agar, xanthgum, pectin, and alginate that are obtained from a variety of sources, including plants, bacteria, and fungi. Meat products, desserts, pet foods, dairy products, ready-to-eat (RTE) meals, sauces, baked goods, candies, and food dressings are the main food categories that use hydrocolloids (154). All living organisms need energy to perform a variety of biological tasks, and most of this energy comes from polysaccharides that are either synthesized or taken as food. Living systems metabolize polysaccharides from numerous sources to produce energy for physiological processes. However, polysaccharides as a source of energy have been mostly associated to be sourced from plants and algae (starch) and animals (glycogen). However, knowledge is lacking regarding linking bacterial derived polysaccharides as a source of energy for the host. Also, there is a shortage of research connecting bacterially produced polysaccharides with potential sources for biological processes such molecular transportation, absorption, digestion, and metabolism.

## Perspectives

### Functional and structural metagenome-based screening could be utilized to search for novel EPS

The proportion of bacteria that can be cultured using conventional culturing techniques is really small compared to the huge bacterial diversity in nature (155). Most unexplored microorganisms that are living in various environments must have evolved various mechanisms to produce novel polysaccharides. Despite of the bacterial diversity and omnipresence, surprisingly limited literature exists about the bacterial polysaccharides, their diversity and function. It is highly likely that bacteria from various habitats would constitute pools of unique polysaccharides that could hold great potential and utilization in the food and feed industry, medical sector, and other manufacturing industries. Since the study of bacterial polysaccharides is partly hindered by the incapability of culturing methods, therefore, metagenome-based techniques should be utilized to dig for and explore novel bacterial polysaccharides. Metagenomic libraries can study the function of diverse bacterial communities like the gut of various animals, forest environments, soil, marine, sediments, and other pristine environments. These metagenomic libraries can further be screened using the functional bioprospecting metagenomics based techniques (156, 157) to select for novel and unique polysaccharides producing clones. These polysaccharides producing clone pools can further be utilized for a variety of purposes including prebiotic and industrial applications.

### Integration of cutting-edge tools for speeding up the discovery and study of bacterial polysaccharides

To understand and harness the potential of bacterial polysaccharides from a wide range of habitats, it is crucial to develop and maintain pure bacterial cultures for identification, isolation, and analysis of polysaccharides. By looking at the diversity and hostility of various bacterial niches, it is vital to comprehend and replicate the naturally occurring environmental conditions for bacteria in a lab. Some researchers have already attempted to retrieve maximum bacterial diversity by replicating their natural environments in lab conditions (158–160). In a recent study, a culture approach was utilized to mimic the plant rhizosphere environment and cultured a portion of the rhizosphere microbiome (161). The portion of the microbiome obtained *via* this mixed culture approach was representative of the function of the original microbiota as it conferred resistance against a pathogenic bacterium like the original microbiome of the rhizosphere. Therefore, such approaches could be utilized to mimic the original habitat of the microbiome to dissect the function and structure of the microbiome.

There are two main obstacles when it comes to culturing microorganisms (bacteria in particular) from an environment. The primary obstacle is the isolation and culturing of a single bacterium from a complex bacterial community. Bacteria are ubiquitous and a huge number of different bacteria constitute a bacterial community in an environment. For instance, around 10^11^ bacteria are present in 1 gram of stool (162), 10^8^ cells inhabit each gram of soil (163), and about 10^5^ cells per ml of surface seawater (164). A solution for the selection of a single bacterium from a complex community is laser guided isolation of a single cell. In this case, single-cell ejection technology is coupled with laser-induced forward transfer (LIFT) techniques for the isolation of bacteria. In previously developed LIFT based techniques, the survival rate of the organisms was lower, however recently a three-layer LIFT system has been developed (165) which has not only increased the survival rate during isolation of the bacteria but has further widened the window of the organisms that could be isolated from a diverse and dense natural environment. Another technique used to enrich a single bacterium population and then explore their functional potential is the use of the iChip technology (166). In the iChip based approach, the environmental sample is initially diluted in a way that aliquots contain a single bacterial cell in a specialized medium. These aliquots are then introduced to the individual wells of iChip. The iChip is then placed back in the original environment (for example, source soil) and after a certain period, the aliquots are again spread onto that specialized media. This technique has been successfully used to isolate antibiotic-producing novel bacterial strains from soil (166).

The secondary obstacle in isolating and culturing the previously uncultured bacteria is their rapid and accurate identification. Although several identification techniques are in place which is either used alone or in combination. However, most of these techniques are laborious and time consuming. On the other hand, matrix assisted laser desorption ionization-time of flight mass spectrometry (MALDI-TOF MS) is a rapid, reliable, and cost-effective method for bacterial identification. MALDI-TOF MS has already been employed as an early identification method in a variety of bacterial and fungal samples like blood cultures, urinary tract infections (UTIs), cerebrospinal fluids, respiratory tract infections and stool samples (167). Therefore, various techniques, such as those mentioned above and others, including NGS-based techniques could be utilized in combination for the culturing and identification of novel bacterial isolates from a variety of environments, where most of the microbial population is still unexplored.

### Polysaccharides-based synthetic microbiome—A futuristic approach for prebiotic intervention

Synthetic microbial communities (SynCom) could be comprised of a group of chosen culturable bacteria that possess the ability to seed in a host's gut and exert health-promoting effects on the host (168). In this sense, SynCom is relatively advantageous in that it can be manipulated by simply adding, eliminating, or substituting one or a few strains to achieve desired functions for improving the host health. SynCom-based approaches have already been used to assess the safety and functionality of corresponding microbial consortia in some diseases (169). This SynCom were tested in alternative hosts such as germ-free mice, rats, or pigs, even though the source organisms of these SynCom were derived from humans (169). So far, various SynCom-based combinations have been used including the famous Altered Schaedler Flora (ASF) (169). In one study, ASF-based SynCom in mice significantly reduced the *C. botulinum* infection mediated death rate compared to the non-treated controls (170). The major limitation of the ASF was its low diversity and poor representation of the overall GM.

Considering the limitations of ASF, various other formulations including the Oligo-MM (murine microbiota) were also tested which comprised twelve members. The Oligo-MM-based SynCom resulted in significant protection against pathogenic microorganisms compared to ASF-based communities (171). The role of SynCom-based communities have also been tested in IBD in mice (172) and it was determined that a single pathogenic bacteria inclusion could lead to IBD condition even in the presence of normal representative flora. This was the foundation finding which led to the idea that the GM has a role in the IBD establishment.

Next-generation sequencing (NGS) based techniques coupled with structural and functional bioprospecting metagenomics can be utilized to explore the vast diversity of unique polysaccharides producing microorganisms. Further, upon identifying those candidate genera, recent advances in culturomics (159) could then be utilized to culture those organisms and could finally be incorporated into various SynCom combinations as probiotics. Special emphasis should be placed on culturing members of the dominantly inhabited phyla but with still negligible cultured members. One example of such phyla is the Verrucomicrobia which is detected to be abundant in a variety of environments including the human gut, but still, the number of culturable organisms from this phylum is insignificant. Various combination approaches should be utilized to culture these unexplored organisms and harness their probiotic and prebiotic potential.

## Author contributions

RK, MS, LS, and P-CL has written a part of the manuscript. IK has designed, written a part of the manuscript, and revised the draft. All authors contributed to the article and approved the submitted version.

## Funding

This study was supported by Universiti Malaysia Sabah-UMSGreat GUG0524-2/2020.

## Conflict of interest

The authors declare that the research was conducted in the absence of any commercial or financial relationships that could be construed as a potential conflict of interest.

## Publisher's note

All claims expressed in this article are solely those of the authors and do not necessarily represent those of their affiliated organizations, or those of the publisher, the editors and the reviewers. Any product that may be evaluated in this article, or claim that may be made by its manufacturer, is not guaranteed or endorsed by the publisher.
